# Characterization of the first complete genome sequence of an *Impatiens necrotic spot orthotospovirus* isolate from the United States and worldwide phylogenetic analyses of INSV isolates

**DOI:** 10.1186/s13104-018-3395-5

**Published:** 2018-05-10

**Authors:** Kaixi Zhao, Paolo Margaria, Cristina Rosa

**Affiliations:** 1 Department of Plant Pathology and Environmental Microbiology, Pennsylvania State University, University Park, PA 16802 USA; 2 Plant Virus Department, Leibniz-Institut DSMZ-Deutsche Sammlung von Mikroorganismen und Zellkulturen GmbH, 38124 Brunswick, Germany

**Keywords:** *Impatiens necrotic spot orthotospovirus*, *Orthotospovirus*, Complete genome sequence, Phylogenetic analysis, Recombination

## Abstract

**Objective:**

*Impatiens necrotic spot orthotospovirus* (INSV) can impact economically important ornamental plants and vegetables worldwide. Characterization studies on INSV are limited. For most INSV isolates, there are no complete genome sequences available. This lack of genomic information has a negative impact on the understanding of the INSV genetic diversity and evolution. Here we report the first complete nucleotide sequence of a US INSV isolate.

**Results:**

INSV-UP01 was isolated from an impatiens in Pennsylvania, US. RT-PCR was used to clone its full-length genome and Vector NTI to assemble overlapping sequences. Phylogenetic trees were constructed by using MEGA7 software to show the phylogenetic relationships with other available INSV sequences worldwide. This US isolate has genome and biological features classical of INSV species and clusters in the Western Hemisphere clade, but its origin appears to be recent. Furthermore, INSV-UP01 might have been involved in a recombination event with an Italian isolate belonging to the Asian clade. Our analyses support that INSV isolates infect a broad plant-host range they group by geographic origin and not by host, and are subjected to frequent recombination events. These results justify the need to generate and analyze complete genome sequences of orthotospoviruses in general and INSV in particular.

**Electronic supplementary material:**

The online version of this article (10.1186/s13104-018-3395-5) contains supplementary material, which is available to authorized users.

## Introduction

Orthotospoviruses cause high economic losses worldwide [1, 2]. Most of the information about orthotospoviruses was acquired by studying the type species *Tomato spotted wilt orthotospovirus* (TSWV) [3–16] and their molecular features are shared among members of the genus. Orthotospoviruses are transmitted by thrips (order: *Thysanoptera*, family: *Thripidae*) in a circulative and propagative manner [17–20]. Four species belonging to the genus *Frankliniella* are INSV vectors [21].

Orthotospoviruses are classified based on nucleocapsid (*N*) amino-acid (aa) sequence identity and serological cross reactivity, plant host range and thrips transmission specificity [22], and are considered as distinct species when their nucleocapsid aa identity is less than 90% [23]. INSV was first designated as TSWV-I strain [24, 25, 26, 27]. INSV glycoproteins are serologically related to TSWV, while the N proteins are serologically unrelated [24, 28].

INSV’s host range includes about 300 plant species [22]. Even though INSV can considerably affect vegetables, its economic importance for vegetables was less than for ornamental plants [29, 30], but in the last few years [21, 31]. INSV importance is increasing in vegetables in Europe and North America.

Characterization studies on INSV are quite limited. Until now, only four complete INSV genomes have been sequenced. The type isolate (NL-07) was reported by van Poelwijk et al. in 1997 and consists of an L and S segments from the Netherlands [32, 33], and an M segment from the US [34] (M74904.1; NC_003625.1, NC_003616.1, NC_003624.1). The M segment from the US was included in the type isolate because it differed by only 4 nucleotides from the M segment of NL-07, whose 700 (out of 5000) nucleotides were sequenced at that time, even though the similarity between the remaining 4300 nucleotides was unknown. Among the three remaining INSV full genome sequences, one is from Italy (DQ425094.1, DQ425095.1, DQ425096.1) and two from China (GQ336989.1, GQ336990.1, GQ336991.1; GU112503.1, GU112504.1, GU112505.1). Isolate GU112505.1 from China contains a non-functional RdRp due to mutation and is missing a portion of the S segment, lowering the number of complete INSV sequences de facto to two. Availability of genomic sequences from different geographic origin is pivotal to understand INSV genetic diversity and evolution, especially considering that orthotospoviruses have a tripartite genome and can reassort. Furthermore, while for other orthotospoviruses like TSWV, the aa sequence of N is sufficiently diverse to confer phylogenetic character, the INSV-N is highly conserved and it is not phylogenetically informative [35–37]. Genetic analysis can be used to characterize the structure of a virus population in relation to a location or host, and to probe the origin in a population and gene flow across time and space. Thus, we suggest that it is important to fully sequence a larger number of INSV genomes, and information gained by doing so will generate understanding of the etiology and aid management of the disease.

## Main text

### Methods

INSV isolate UP01 was found in a commercial greenhouse in Pennsylvania in July 2014, in an impatiens showing ringspots symptom, acquired complying with Penn State institutional guidelines. The plant was initially tested for TSWV, INSV, *Tobacco mosaic virus* (TMV), and *Cucumber mosaic virus* (CMV) by ImmunoStrip^®^ assays (Agdia, Elkhart, IN, USA), and found to be infected only with INSV. Following four passages by mechanical inoculation from single lesions on *Nicotiana benthamiana*, the virus species was confirmed by ELISA assay (Agdia, Elkhart, IN, USA). Mechanical inoculations were used to assess the partial INSV isolate host range. Inoculated plants were maintained in growth chambers at 25 °C with 16 h photoperiod for symptom development. All inoculated plants were tested by ELISA for INSV.

This isolate was transmitted from *Emilia sonchifolia* to *E. sonchifolia* by *Frankliniella occidentalis* (Western flower thrips, WFT) to verify its vector transmissibility. Thrips transmission experiments ([21], with modifications) were conducted with symptomatic leaves from infected *E. sonchifolia* as virus source. First-instar larvae (12 h old) of WFT were given a 24 h acquisition access period and then reared on virus-free green bean pods until adulthood. These adult thrips were given a 48-h inoculation access period to 2 weeks old *E. sonchifolia* seedlings (20 thrips per plant). This experiment was repeated twice. Inoculated plants were maintained in a growth chamber (25 °C, 16 h photoperiod) for symptom development and then were tested by ELISA.

Transient agroinfiltration was used to test the functionality of the INSV NSs protein as silencing suppressor according to previous protocols [12, 38]. Briefly, full-length UP01 NSs was cloned into pBin61 vector and transiently expressed through agroinfiltration together with pBin-GFP in 16C *N. benthamiana*. Vector only (pBin61) and pBin61-p19, both together with pBin-GFP, were used as negative control and positive control, respectively. GFP expression of agroinfiltrated plants was checked with UV light 3 days post-agroinfiltration.

Total RNA was extracted from systemically infected *N. benthamiana* leaves using the Spectrum™ Plant-Total RNA Kit (Sigma-Aldrich, St. Louis, MO, USA), following the manufacturer’s directions. Reverse transcription was performed using Superscript IV reverse transcriptase (Invitrogen, Grand Island, NY, USA), random primers and 500–1000 ng of RNA as template. Overlapping amplicons were obtained by PCRs with gene-specific primers designed on conserved regions of available INSV isolates (Additional file 1) and the Q5 High Fidelity PCR Kit (NEB, Ipswich, MA, USA), followed by 5 min adenylation at 72 °C using GoTaq DNA Polymerase (Promega, Madison, WI, USA). PCR products were cloned into pGEM-T Easy vector (Promega, Madison, WI, USA) and sequenced at the PSU Genomic Core Facility by Sanger sequencing. Overlapping sequences were assembled using Vector NTI software (Invitrogen, Grand Island, NY, USA).

Phylogenetic trees were constructed by neighbor-joining method [39] using MEGA7 software [40], with 1000 bootstrap replicates. Percentages of pairwise identity among the aligned nucleotide and protein sequences were calculated using MatGAT v.2.03 [41]. Putative reassortment and recombination events were predicted by Recombination Detection Program (RDP4 v.4.80) [42] using several algorithms on the MUSCLE alignment file of concatenated full-length genome sequences, created with MEGA7.

### Results

#### Symptoms, hosts and vector

INSV-UP01 produced typical INSV symptoms of chlorotic blotches and mottling on local leaves of *N. benthamiana* at 4–5 days post-inoculation and curling of newly emerged leaves; and chlorotic spots, mosaic and mottling on the systemic leaves of *E. sonchifolia*. On both hosts the isolate produced occasional ringspots on the systemic leaves. The isolate was successfully transmitted by *F. occidentalis*. *N. benthamiana*, *N. tabacum*, impatiens, pepper, *Datura stramonium* and *E. sonchifolia*, could be infected with UP01, but not *Arabidopsis thaliana*.

#### Genome organization

The three genomic INSV RNA segments were 8776, 4975 and 3010 nt in length, respectively (Additional files 2, 3, 4, NCBI accession numbers MH171172–MH171174). The L segment was predicted to contain an ORF of 8598 nt in position 8747-150 and to encode a putative RdRp protein of 2865 aa, in the negative sense. The M segment encoded the putative NSm protein in the viral sense in position 86-997 and the putative Gn/Gc protein precursor in the complementary sense in position 4805-1473, separated by an intergenic region of 475 nt. The M segment had 85 and 170 nt in its 5′ and 3′ UTR, respectively. The S segment encoded a putative N protein in position 2861-2073, and a putative NSs protein in position 80-1429, with an intergenic AU-rich region of 643 nt. Multifunctional properties of the NSs protein have been shown for orthotospoviruses [14]. Since the NSs of TSWV has been demonstrated to function as silencing suppressor [12, 43–45], we performed *in planta* transient *Agrobacterium tumefaciens* silencing suppression assays [38] to test this activity for UP01 and demonstrated that UP01 NSs is a strong silencing suppressor (Additional file 5).

#### Conserved motifs

Several amino acid substitutions distributed along the whole RdRp protein sequence were observed between UP01 and other INSV isolates (Additional file 6). UP01′s RdRp shared 97.6 and 97.2% nt identity, and 98.8 and 98.4% aa identity with NL-07 (X93216.1) and DQ425094.1, respectively (Additional file 7), and showed motifs conserved in the RdRp of this genus: motif A (DXXKW), motif B (QGXXXXXSS), motif C (SSD), motif E (EXXS) [46], motif F2 (KXQRTK) and motif F3 (DREIY) [47]. Motif F1 (TDF), [48, 49] absent in all sequenced INSVs, was also not present in UP01. UP01 NSm predicted protein sequence had the ‘D-motif’ [50], which is a conserved region in the majority of viral movement proteins belonging to the ‘30K superfamily’ and ‘the P/D-L-X motif’ [51], ‘DSRK motif’ and ‘HH motif’, which play essential roles in the subcellular distribution and tubule formation of TSWV NSm protein [52].

#### A recombination event in the L segment is predicted among INSV isolates

Analysis of putative reassortment/recombination events using INSV concatenated full-length genome sequences predicted the occurrence of a recombination event involving isolates UP01, NL-07 and the Italian isolate (Additional file 8). The event involved the L segment and was predicted by different algorithms with significance level set at P ≤ 0.05.

### Discussion

**Fig. 1:**
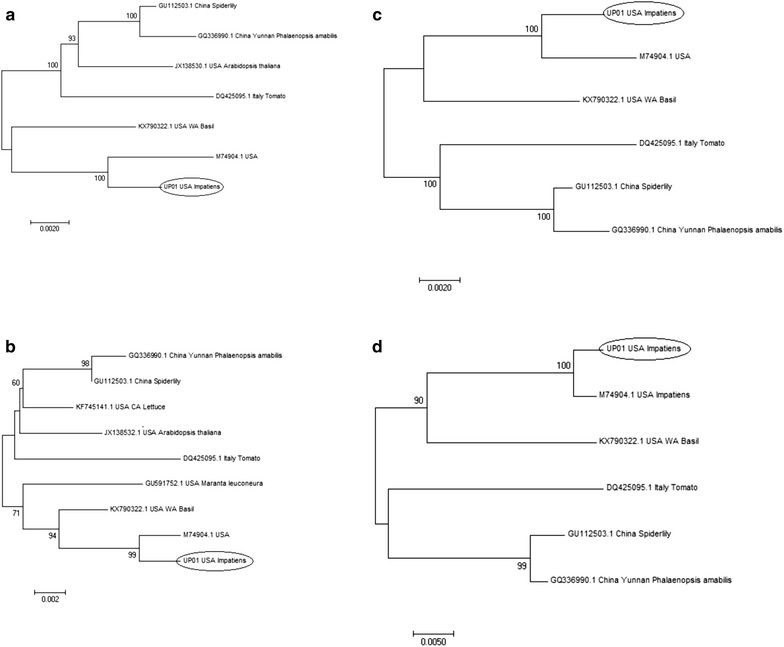
Neighbor-joining phylogenetic tree derived from the alignment of the **a** Gn/Gc polyprotein coding sequence (cds); **b** NSm protein cds; **c** M segment nucleotide sequence and **d** M segment intergenic region nucleotide sequence of different INSV isolates. Bootstrap values were derived from 1000 bootstrap replicates. Accession numbers and plant host species of the sequences are shown in the figure. Scale bar represents number of substitutions per site

**Fig. 2:**
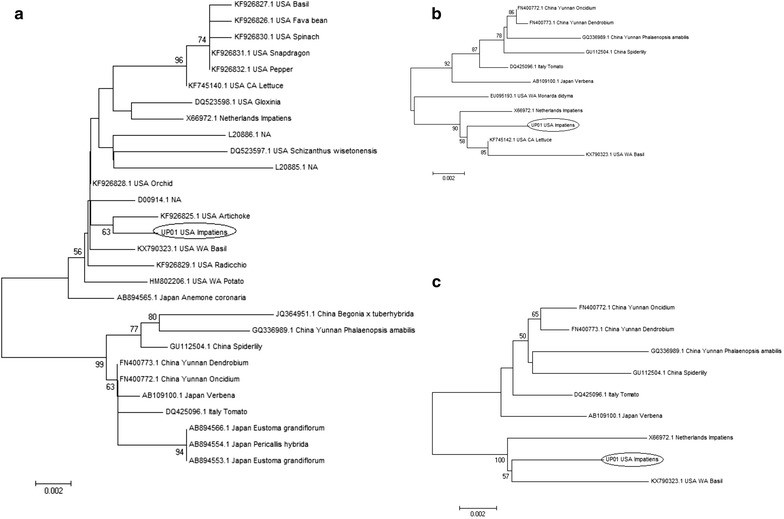
Neighbor-joining phylogenetic tree derived from the alignment of **a** N protein cds; **b** NSs protein cds and **c** S segment nucleotide sequence of different INSV isolates. Bootstrap values were derived from 1000 bootstrap replicates. Accession numbers and plant host species of the sequences are shown in the figure. Scale bar represents number of substitution per site

**Fig. 3:**
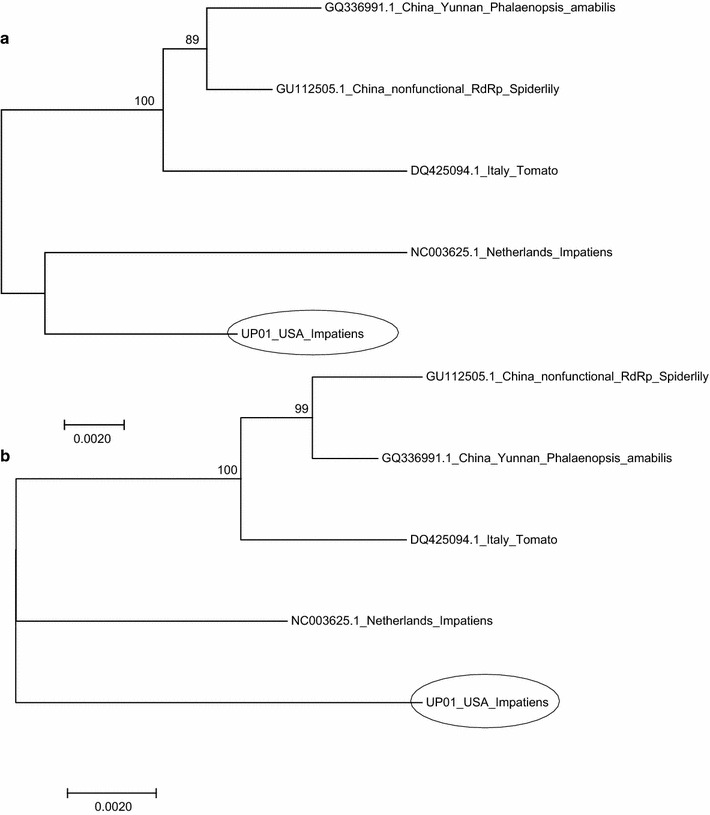
Neighbor-joining phylogenetic tree derived from the alignment of breakpoint for recombination different INSV isolates. **a** Sequences on L segment from 1 to 2849 nt and **b** sequences on L segment from 2850 to 8690 nt. Bootstrap values were derived from 1000 bootstrap replicates. Scale bar represents number of substitution per site. Accession numbers and plant host species of the sequences are shown in the figure

UP01 is consistently placed into the same Western Hemisphere clade with other US isolates and NL-07, and is more distantly related to isolates in the Asian clade, where the Italian isolate also belongs (Figs. 1, 2, 3). As indicated by Elliott et al. [36] and Nekoduka et al. [37], our result confirms that INSV isolates do not group phylogenetically based on host species (Figs. 1, 2; Additional file 9).

UP01 RdRp ORF is overall more related to NL-07 than to other isolates (Additional file 8) but it shares different degrees of similarities with all isolates based on the region of the RdRp examined, suggesting a possible recombination event for this segment involving the region of 2850-8690. The resolution of the RdRp phylogeny is penalized by having only 5 sequences available.

Phylogenetic analyses of the M segment and its two ORFs (Gn/Gc and NSm) (Fig. 1a–c) and IGR (Fig. 1d) show again that INSV isolates are divided into Western Hemisphere and Asian clades, with UP01 in the Western Hemisphere clade, and the isolates from Italy and Asia in the Asian clade. In the Asian clade are unexpectedly grouped also one *A. thaliana* (NSm JX138532.1, Gn/Gc JX138530.1) (Fig. 1a, b) and one lettuce isolates (KF745141.1) (Fig. 1b) from the US, suggesting that these isolates might be of European/Asian origin and have been introduced recently in the US. While this segment is better represented, still not many sequences are available to resolve some of the phylogenetic relationships for members of the two clades.

Phylogenetic analyses using N protein nucleotide sequences (Fig. 2a) indicate that UP01 grouped in the Western Hemisphere clade. This clade contains isolates from the US and the Netherlands, but also one isolate from Japan (AB894565.1), again indicating that INSV was probably introduced into different regions via import of infected plant material. UP01N protein shares very high aa identity with other INSV isolates (Additional file 10).

The division into Western Hemisphere and Asian clade is also congruent when looking at the phylogenetic analyses of the S segment (Fig. 2c), where UP01 belongs to the Western Hemisphere clade and is distantly related to the Chinese isolate (GU112504.1). But while for the M segment UP01 is closely related to the reference sequence (M74904.1, NC_003616.1), with whom it shares a more recent origin (bootstrap value > 90%), and it is less related with USA WA Basil isolate (KX790322.1) (bootstrap value > 90%) (Fig. 1c), the phylogenetic study of the S segment (Fig. 2c) revealed that UP01 is more related to the USA WA basil isolate than to the reference sequence. This observation, for the first time, questions combining in a reference genome sequences that superficially seem to belong to the same isolate, but that could belong to distinct clades, when analyzed using a larger number of sequences. An alternative explanation to our result could indicate a reassortment event between isolates from in different geographic regions that led to the emergence of the reference genome. Interestingly, the USA CA lettuce isolate SV-L1 (NSs KF745142.1 and N KF745140.1, respectively) that was isolated from an INSV outbreak in Coastal California clustered with other US isolates when its NSs (Fig. 2b) and N ORFs (Fig. 2a) were analyzed, but its NSm (KF745141.1) sequence grouped with the Asian clade with high bootstrap support (Fig. 1b), indicating a possible reassortment or recombination event. Unfortunately, the Gn/Gc sequences for these isolates are not available to support these hypotheses.

The phylogenetic analysis of the N protein (Fig. 2a) is the one for which more sequences are available, and highlights how having a large number of sequences can resolve better the INSV phylogeny and can be epidemiologically informative. In fact, in the case of the INSV sequences reported in a recent outbreak in lettuce in Costal California [31], phylogeny shows that all lettuce strains responsible for the outbreak were identical or highly related, but they differed from isolates found in the surrounding weeds and crops.

Result of the analysis of putative reassortment/recombination suggests that a recombination event involving UP01 might have happened. As mentioned above, phylogenetic analysis also supports the predicted recombination event (Fig. 3) and further confirms the occurrence of genetic exchanges in the evolution of orthotospoviruses. Reassortment is also biologically important because it could result in new resistant-breaking strains [45, 53, 54] or emergence of new viruses [55].

## Limitations

Additional complete genome sequences from the INSV outbreak in Coastal California would be needed to confirm reassortment and recombination events between INSV isolates.

## Additional files

**Additional file 1.** List of primers. The table contains the list of primers used for RT-PCR and Sanger sequencing of isolate UP01, and includes the segment name, sequence from 5′ to 3′, and name for each of the primers listed.

**Additional file 2.** Sequence of INSV UP01 L segment in fasta format.

**Additional file 3.** Sequence of INSV UP01 M segment in fasta format.

**Additional file 4.** Sequence of INSV UP01 S segment in fasta format.

**Additional file 5.** Bioassays for silencing suppression activity of UP01 NSs allelic variant. Full-length UP01 NSs was cloned into pBin61 vector and transiently expressed through agroinfiltration together with pBin-GFP in 16C transgenic *Nicotiana benthamiana*. Vector only (pBin61) andpBin61-p19 were used as negative control and positive control, respectively. Pictures were taken by using a hand-held UV light 3 days post-agroinfiltration.

**Additional file 6.** Alignment of INSV RdRp amino acid sequences. Figure shows the amino acid substitutions distributed along the 2221–2760 amino acids of the Query: UP01 and Sbjct: NC_003625.1 RdRp protein sequences.

**Additional file 7.** (a) pairwise comparison of RdRp predicted amino acid sequences and (b) pairwise comparison of RdRp nucleotide sequences. The table contains the RdRp nt pairwise comparison among INSV isolates listed in the first column. Values in blue cells indicate percentage identity. INSV isolates are identified by their accession numbers in NCBI.

**Additional file 8.** Putative recombination event among full-length INSV isolates of different geographic origin, predicted by the RDP software. Analysis of putative reassortment/recombination events using INSV concatenated full-length genome sequences predicted a possible recombination event involving isolates UP01, NL-07 (from the Netherlands) and the Italian isolate. The event involved the L segment and was predicted by different algorithms, with significance level set at P ≤ 0.05.

**Additional file 9.** Neighbor-joining phylogenetic tree derived from the alignment of INSV RdRp coding sequence (cds) of different INSV isolates. Bootstrap values were derived from 1000 bootstrap replicates. Accession numbers and plant host species of the sequences are shown in the figure.

**Additional file 10.** Pairwise comparison of N protein predicted amino acid sequences. Table contains the N protein amino acid pairwise comparison among INSV isolates listed in the first column. Values in blue cells indicate percentage identity. Values in green cells indicate percentage similarity. INSV isolates are identified by their accession numbers in NCBI.

## Abbreviations

INSV: *Impatiens necrotic spot orthotospovirus*
TSWV: *Tomato spotted wilt orthotospovirus*
aa: amino-acid
RDP: Recombination Detection Program
UTR: untranslated region
RdRp: RNA-dependent RNA polymerase
IGR: intergenic region
ORF: open reading frame

## References

[1] Bishop DHL, Shope RE (1979). Bunyaviridae: comprehensive virology.

[2] Plyusnin A, Elliott RM (2011). Bunyaviridae: molecular and cellular biology.

[3] de Haan P, Wagemakers L, Peters D, Goldbach R (1990). The S RNA segment of tomato spotted wilt virus has an ambisense character. J Gen Virol.

[4] German TL, Ullman DE, Moyer JW (1992). Tospoviruses: diagnosis, molecular biology, phylogeny, and vector relationships. Annu Rev Phytopathol.

[5] Wijkamp I, van Lent J, Kormelink R, Goldbach R, Peters D (1993). Multiplication of tomato spotted wilt virus in its insect vector, *Frankliniella occidentalis*. J Gen Virol.

[6] Kormelink R, Storms M, van Lent J, Peters D, Goldbach R (1994). Expression and subcellular location of the NSm protein of tomato spotted wilt virus (TSWV), a putative viral movement protein. Virology.

[7] Stevens MR, Lamb EM, Rhoads DD (1995). Mapping the Sw-5 locus for tomato spotted wilt virus resistance in tomatoes using RAPD and RFLP analyses. Theor Appl Genet.

[8] Goldbach R, Peters D (1996). Molecular and biological aspects of tospoviruses in the Bunyaviridae.

[9] Bandla MD, Campbell LR, Ullman DE, Sherwood JL (1998). Interaction of tomato spotted wilt tospovirus (TSWV) glycoproteins with a thrips midgut protein, a potential cellular receptor for TSWV. Phytopathology..

[10] Richmond KE, Chenault K, Sherwood JL, German TL (1998). Characterization of the nucleic acid binding properties of tomato spotted wilt virus nucleocapsid protein. Virology.

[11] Adkins S (2000). Tomato spotted wilt virus—positive steps towards negative success. Mol Plant Pathol.

[12] Takeda A, Sugiyama K, Nagano H (2002). Identification of a novel RNA silencing suppressor, NSs protein of Tomato spotted wilt virus. FEBS Lett.

[13] Hogenhout SA, Ammar ED, Whitfield AE, Redinbaugh MG (2008). Insect vector interactions with persistently transmitted viruses. Annu Rev Phytopathol.

[14] Geerts-Dimitriadou C, Lu YY, Geertsema C, Goldbach R, Kormelink R (2012). Analysis of the Tomato spotted wilt virus ambisense S RNA-encoded hairpin structure in translation. PLoS ONE.

[15] Murphy FA, Fauquet CM, Bishop DH, Ghabrial SA, Jarvis A, Martelli GP, Summers MD (2012). Virus taxonomy: classification and nomenclature of viruses.

[16] Hedil M, Hassani-Mehraban A, Lohuis D, Kormelink R (2014). Analysis of the AU rich hairpin from the intergenic region of tospovirus S RNA as target and inducer of RNA silencing. PLoS ONE.

[17] Amin PW, Reddy DVR, Ghanekar AM (1981). Transmission of tomato spotted wilt virus, the causal agent of bud necrosis of peanut, by *Scirtothrips dorsalis* and *Frankliniella schultzei*. Plant Dis.

[18] Ullman DE, Meideros R, Campbell LR, Whitfield AE, Sherwood JL, German TL (2002). Thrips as vectors of tospoviruses. Adv Bot Res.

[19] Whitfield AE, Ullman DE, German TL (2005). Tospovirus–thrips interactions. Annu Rev Phytopathol.

[20] Rotenberg D, Jacobson AL, Schneweis DJ (2015). Thrips transmission of tospoviruses. Curr Opin Virol.

[21] Naidu RA, Deom CM, Sherwood JL (2005). Expansion of the host range of *Impatiens necrotic spot virus* to peppers. Plant Health Prog Online..

[22] Pappu HR, Jones RAC, Jain RK (2009). Global status of tospovirus epidemics in diverse cropping systems: successes achieved and challenges ahead. Virus Res.

[23] King AM, Lefkowitz E, Adams MJ, Carstens EB (2011). Virus taxonomy: ninth report of the international committee on taxonomy of viruses.

[24] Law MD, Moyer JW (1990). A tomato spotted wilt-like virus with a serologically distinct N protein. J Gen Virol.

[25] De Avila AC, De Haan P, Kitajima EW, Kormelink R, Resende RDO, Goldbach RW, Peters D (1992). Characterization of a distinct isolate of Tomato spotted wilt virus (TSWV) from Impatiens sp. in the Netherlands. Antonio Carlos de Avila..

[26] Jan F, Chen T, Yeh S, Huang HC, Acharya SN (2003). Occurrence, importance, taxonomy, and control of thrips-borne tospoviruses. Advances in plant disease management.

[27] Lin YH, Chen TC, Hsu HT, Liu FL, Chu FH, Chen CC, Yeh SD (2005). Serological comparison and molecular characterization for verification of Callalily chlorotic spot virus as a new tospovirus species belonging to Watermelon silver mottle virus serogroup. Phytopathology..

[28] Vaira AM, Roggero P, Luisoni E, Masenga V, Milne RG, Lisa V (1993). Characterization of two tospoviruses in Italy: Tomato spotted wilt and impatiens necrotic spot. Plant Pathol.

[29] Daughtrey ML, Jones RK, Moyer JW, Daub ME, Baker JR (1997). Tospoviruses strike the greenhouse industry: INSV has become a major pathogen on flower crops. Plant Dis.

[30] Turina M, Tavella L, Ciuffo M (2012). Tospoviruses in the Mediterranean area. Adv Virus Res.

[31] Kuo YW, Gilbertson RL, Turini T, Brennan EB, Smith RF, Koike ST (2014). Characterization and epidemiology of outbreaks of *Impatiens necrotic spot virus* on lettuce in coastal California. Plant Dis.

[32] van Poelwijk F, Prins M, Goldbach R (1997). Completion of the *Impatiens necrotic spot virus* genome sequence and genetic comparison of the L proteins within the family Bunyaviridae. J Gen Virol.

[33] de Haan P, de Avila AC, Kormelink R, Westerbroek A, Gielen JJ, Peters D, Goldbach R (1992). The nucleotide sequence of the S RNA of *Impatiens necrotic spot virus*, a novel tospovirus. FEBS Lett.

[34] Law MD, Speck J, Moyer JW (1992). The M RNA of impatiens necrotic spot Tospovirus (Bunyaviridae) has an ambisense genomic organization. Virology.

[35] Abad J, New S, Speck J, Ochoa-Corona F, Moyer J (2005). Phylogenetics of *Impatiens necrotic spot virus* 1981 (INSV) based on optimal sequence alignment (OSA) of the N, NsM and L protein genes. Phytopathology..

[36] Elliott DR, Lebas BS, Ochoa-Corona FM, Tang J, Alexander BJ (2009). Investigation of *Impatiens necrotic spot virus* outbreaks in New Zealand. Aus Plant Pathol.

[37] Nekoduka S, Kobayashi K, Fuji SI, Okuda M, Sano T (2015). Molecular epidemiology of *Impatiens necrotic spot virus* on greenhouse ornamental plants in a local area of Japan. J Gen Plant Pathol.

[38] Margaria P, Ciuffo M, Pacifico D, Turina M (2007). Evidence that the nonstructural protein of tomato spotted wilt virus is the avirulence determinant in the interaction with resistant pepper carrying the Tsw gene. Mol Plant Microbe Interact.

[39] Saitou N, Nei M (1987). The neighbor-joining method: a new method for reconstructing phylogenetic trees. Mol Biol Evol.

[40] Kumar S, Stecher G, Tamura K (2016). MEGA7: molecular evolutionary genetics analysis version 7.0 for bigger datasets. Mol Biol Evol.

[41] Campanella JJ, Bitincka L, Smalley J (2003). MatGAT: an application that generates similarity/identity matrices using protein or DNA sequences. BMC Bioinform.

[42] Martin DP, Murrell B, Golden M, Khoosal A, Muhire B (2015). RDP4: detection and analysis of recombination patterns in virus genomes. Virus Evol.

[43] Bucher E, Sijen T, de Haan P, Goldbach R, Prins M (2003). Negative-strand tospoviruses and tenuiviruses carry a gene for a suppressor of gene silencing at analogous genomic positions. J Virol.

[44] Kormelink R, Garcia ML, Goodin M, Sasaya T, Haenni AL (2011). Negative-strand RNA viruses: the plant-infecting counterparts. Virus Res.

[45] Hedil M, Sterken MG, de Ronde D, Lohuis D, Kormelink R (2015). Analysis of tospovirus NSs proteins in suppression of systemic silencing. PLoS ONE.

[46] Müller R, Poch O, Delarue M, Bishop DHL, Bouloy M (1994). Rift valley fever virus L segment: correction of the sequence and possible functional role of newly identified regions conserved in RNA-dependent polymerases. J Gen Virol.

[47] Bag S, Druffel KL, Pappu HR (2010). Structure and genome organization of the large RNA of Iris yellow spot virus (genus Tospovirus, family Bunyaviridae). Adv Virol.

[48] Chen TC, Li JT, Fan YS, Yeh YC, Yeh SD, Kormelink R (2013). Molecular characterization of the full-length L and M RNAs of Tomato yellow ring virus, a member of the genus Tospovirus. Virus Genes.

[49] Meng J, Liu P, Zhu L, Zou C, Li J, Chen B (2015). Complete genome sequence of Mulberry vein banding associated virus, a new tospovirus infecting mulberry. PLoS ONE.

[50] Mushegian AR, Koonin EV (1993). Cell-to-cell movement of plant viruses. Adv Virol.

[51] Silva MS, Martins CRF, Bezerra IC, Nagata T, De Avila AC, Resende RDO (2001). Sequence diversity of NS M movement protein of tospoviruses. Adv Virol.

[52] Li W, Lewandowski DJ, Hilf ME, Adkins S (2009). Identification of domains of the Tomato spotted wilt virus NSm protein involved in tubule formation, movement and symptomatology. Virology.

[53] Margaria P, Ciuffo M, Rosa C, Turina M (2015). Evidence of a tomato spotted wilt virus resistance-breaking strain originated through natural reassortment between two evolutionary-distinct isolates. Virus Res.

[54] Qiu W, Moyer JW (1991). Tomato spotted wilt tospovirus adapts to the TSWV N gene-derived resistance by genome reassortment. Phytopathology.

[55] Webster CG, Frantz G, Reitz SR, Funderburk JE, Mellinger HC, McAvoy E, Daughtrey ML (2015). Emergence of groundnut ringspot virus and tomato chlorotic spot virus in vegetables in Florida and the southeastern United States. Phytopathology.

