# Supplementary material for: Characterization of the first complete genome sequence of an Impatiens necrotic spot orthotospovirus isolate from the United States and worldwide phylogenetic analyses of INSV isolates
Source: BMC Res Notes. 2018 May 10;11:288. doi: 10.1186/s13104-018-3395-5 (PMC5946465; doi:10.1186/s13104-018-3395-5)
Supplement: Supplementary file 1 — Additional file 1. List of primers. The table contains the list of primers used for RT-PCR and Sanger sequencing of isolate UP01, and includes the segment name, sequence from 5′ to 3′, and name for each of the primers listed. [file 13104_2018_3395_MOESM1_ESM.docx]

| **Additional file 1**: Primers used for RT-PCR and sequencing of isolate UP01 | | | |
| --- | --- | --- | --- |
|  |  |  |  |
| **Segment** | **Primer name** | **Sequence (5'-3')** |  |
|  |  |  |  |
| S | J13 | CCGGATCCAGAGCAATTGTGTC | |
|  | INSV-S1410rev | TTCACCTGCAGCATAGTCAA | |
|  |  |  |  |
|  | INSV-S1330for | CAATGTCTTATTTGGATAGC | |
|  | INSV-S2250rev | TGCAGATATACTAAAGGCTTG | |
|  |  |  |  |
|  | INSV-S2190for | TGTAGTGGTCCATTGCAGCA | |
|  |  |  |  |
|  |  |  |  |
| M | J13 | CCGGATCCAGAGCAATTGTGTC | |
|  | INSV-M1275rev | CTGGCCGGAGCCTTATTTATTTAC | |
|  |  |  |  |
|  | INSV-M926for | GAGAAATCTGTTGAAGAAGAGG | |
|  | INSV-M1980rev | GTCAACATACCAGATAGCAGT | |
|  |  |  |  |
|  | INSV-M1910for | GGTTAGAAGTGCAGAACATTG | |
|  | INSV-M3187rev | CTGGTGACACATTGGGATTG | |
|  |  |  |  |
|  | INSV-M2897for | CAACAAACTTCTGAGCAGTGC | |
|  | INSV-M4252rev | CTAATGGCACAGTCTCTCTC | |
|  |  |  |  |
|  | INSV-M4023for | GCACAATAGGTTATGCTTACC | |
|  | INSV-M4946rev | CATACAACAGATCAAACTC | |
|  |  |  |  |
|  | INSV-M4760for | CGCCCCTTTCAACATGAATC | |
|  |  |  |  |
|  |  |  |  |
| L | J13 | CCGGATCCAGAGCAATTGTGTC | |
|  |  |  |  |
|  | INSV-L-2for | GCAGAGCAATCAGGCACAACTAAAATC | |
|  | INSV-L487rev | GAGATATCAGAGTGCCTGGAAGG | |
|  |  |  |  |
|  | INSV-L75for | GGCACTATTTGCTCTTTCAGTTG | |
|  | INSV-L1296rev | CGGGTTTAGGGAAACTGTTAGC | |
|  |  |  |  |
|  | INSV-L1218for | CAACATCAACAACATTGACTTG | |
|  | INSV-L2977rev | AATTTGGTGCTGAAGGATGTG | |
|  |  |  |  |
|  | INSV-L2859for | CCTTCTACAACTGGTATCCA | |
|  | INSV-L4770rev | TGACCCTTCAGAGGCCATAT | |
|  |  |  |  |
|  | INSV-L4625for | CAGAAGCTGACCACTTAGATTG | |
|  | INSV-L6524rev | CTAAAGGTTTGATGGTTGCTG | |
|  |  |  |  |
|  | INSV-L6488for | TTAGTCATGTACCTGTCAGCA | |
|  | INSV-L7740rev | TCCAACACAGATTGCTCATTC | |
|  |  |  |  |
|  | INSV-L7600for | CCCATTAAATCTATCAGCAAT | |
|  |  |  |  |
| Sequencing | |  |  |
|  | INSV-L3575seq | CAAGGAACCTTTCAGAGGATGTG | |
