# Supplementary figures and images for: Characterization of the first complete genome sequence of an Impatiens necrotic spot orthotospovirus isolate from the United States and worldwide phylogenetic analyses of INSV isolates
Source: BMC Res Notes. 2018 May 10;11:288. doi: 10.1186/s13104-018-3395-5 (PMC5946465; doi:10.1186/s13104-018-3395-5)

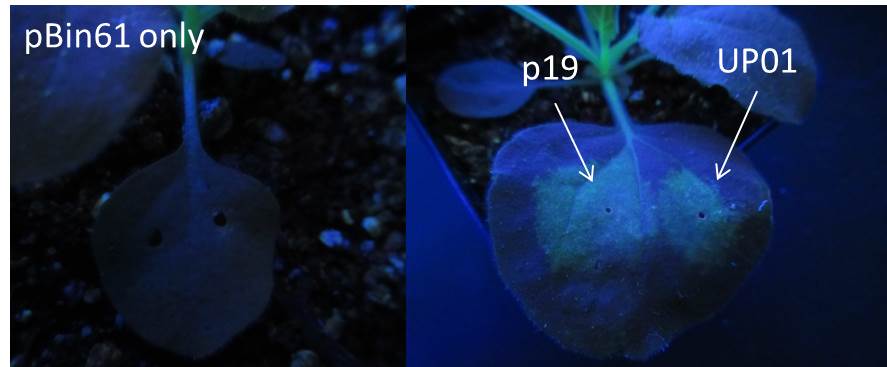

Supplement: Supplementary file 5 — Additional file 5. Bioassays for silencing suppression activity of UP01 NSs allelic variant. Full-length UP01 NSs was cloned into pBin61 vector and transiently expressed through agroinfiltration together with pBin-GFP in 16C transgenic Nicotiana benthamiana. Vector only (pBin61) andpBin61-p19 were used as negative control and positive control, respectively. Pictures were taken by using a hand-held UV light 3 days post-agroinfiltration. [file 13104_2018_3395_MOESM5_ESM.jpg]

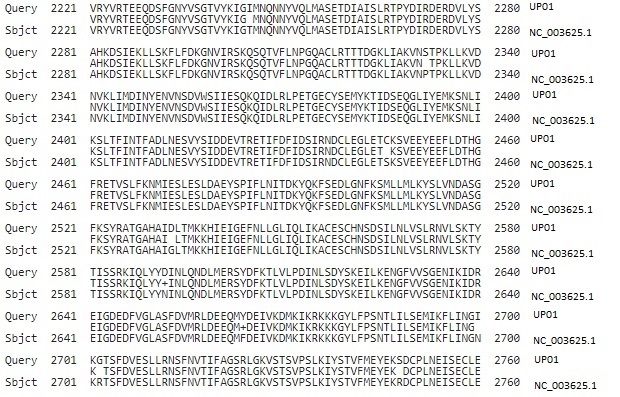

Supplement: Supplementary file 6 — Additional file 6. Alignment of INSV RdRp amino acid sequences. Figure shows the amino acid substitutions distributed along the 2221–2760 amino acids of the Query: UP01 and Sbjct: NC_003625.1 RdRp protein sequences. [file 13104_2018_3395_MOESM6_ESM.jpg]

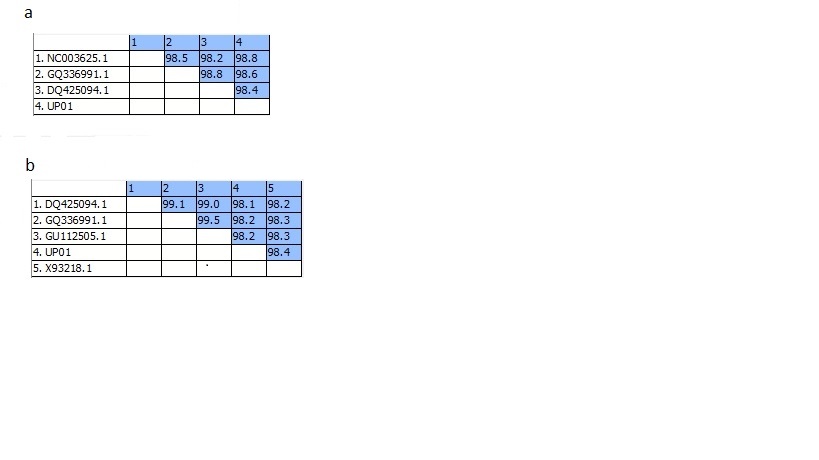

Supplement: Supplementary file 7 — Additional file 7. (a) pairwise comparison of RdRp predicted amino acid sequences and (b) pairwise comparison of RdRp nucleotide sequences. The table contains the RdRp nt pairwise comparison among INSV isolates listed in the first column. Values in blue cells indicate percentage identity. INSV isolates are identified by their accession numbers in NCBI. [file 13104_2018_3395_MOESM7_ESM.jpg]

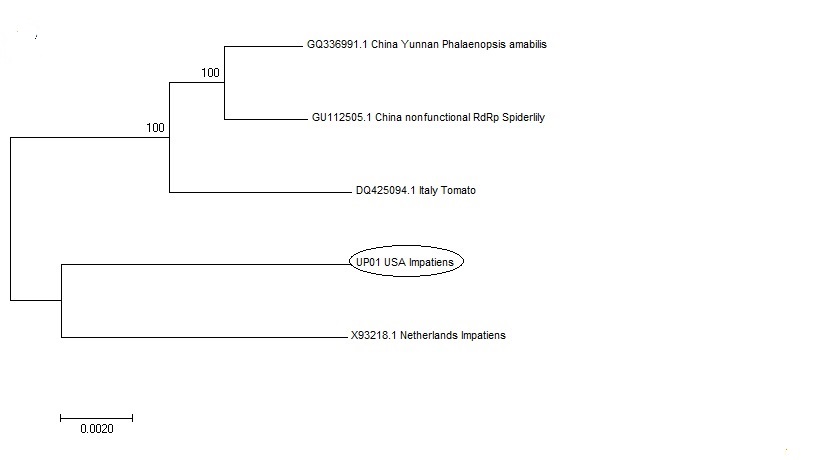

Supplement: Supplementary file 9 — Additional file 9. Neighbor-joining phylogenetic tree derived from the alignment of INSV RdRp coding sequence (cds) of different INSV isolates. Bootstrap values were derived from 1000 bootstrap replicates. Accession numbers and plant host species of the sequences are shown in the figure. [file 13104_2018_3395_MOESM9_ESM.jpg]

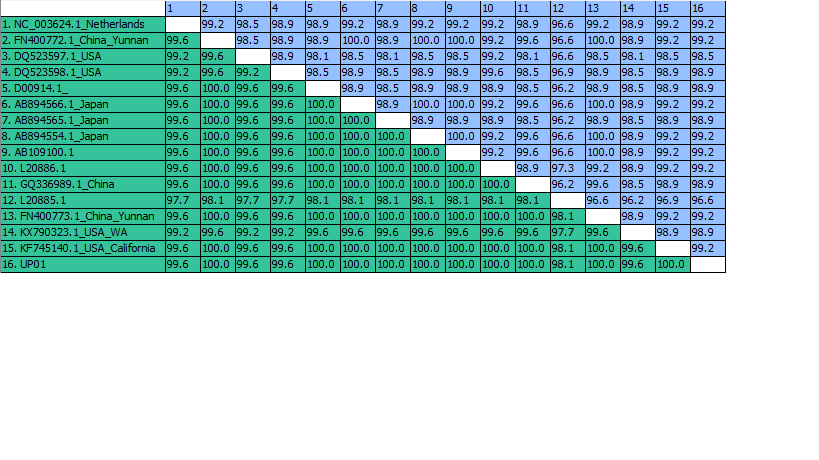

Supplement: Supplementary file 10 — Additional file 10. Pairwise comparison of N protein predicted amino acid sequences. Table contains the N protein amino acid pairwise comparison among INSV isolates listed in the first column. Values in blue cells indicate percentage identity. Values in green cells indicate percentage similarity. INSV isolates are identified by their accession numbers in NCBI. [file 13104_2018_3395_MOESM10_ESM.jpg]
