# Supplementary material for: Characterization of the first complete genome sequence of an Impatiens necrotic spot orthotospovirus isolate from the United States and worldwide phylogenetic analyses of INSV isolates
Source: BMC Res Notes. 2018 May 10;11:288. doi: 10.1186/s13104-018-3395-5 (PMC5946465; doi:10.1186/s13104-018-3395-5)
Supplement: Supplementary file 8 — Additional file 8. Putative recombination event among full-length INSV isolates of different geographic origin, predicted by the RDP software. Analysis of putative reassortment/recombination events using INSV concatenated full-length genome sequences predicted a possible recombination event involving isolates UP01, NL-07 (from the Netherlands) and the Italian isolate. The event involved the L segment and was predicted by different algorithms, with significance level set at P ≤ 0.05. [file 13104_2018_3395_MOESM8_ESM.docx]

| **Additional file 8**:Putative reassortment/recombination event among full-length INSV isolates of different geographic origin, predicted by RDP software v. 4.80 | | | | | | | | | | | | |
| --- | --- | --- | --- | --- | --- | --- | --- | --- | --- | --- | --- | --- |
|  |  |  |  |  |  |  |  |  |  |  |  |  |
|  | Recombinant * |  | NL-07 |  |  |  |  |  |  |  |  |  |
|  | Event |  | 1 |  |  |  |  |  |  |  |  |  |
|  | Initial Breakpoint Position | | 2850 |  |  |  |  |  |  |  |  |  |
|  | Ending Breakpoint Position | | 8690 |  |  |  |  |  |  |  |  |  |
|  | Genomic segment |  | L |  |  |  |  |  |  |  |  |  |
|  | Minor parent |  | Italian isolate |  |  |  |  |  |  |  |  |  |
|  | Major parent |  | UP01 |  |  |  |  |  |  |  |  |  |
|  | P-values determined | RDP | 0.00483 |  |  |  |  |  |  |  |  |  |
|  | by different | Geneconv | 0.0611 |  |  |  |  |  |  |  |  |  |
|  | algorithms | MaxChi | 0.00000118 |  |  |  |  |  |  |  |  |  |
|  |  | Bootscan | 0.000653 |  |  |  |  |  |  |  |  |  |
|  |  | Chimaera | 0.0371 |  |  |  |  |  |  |  |  |  |
|  |  | SiScan | 4.62E-16 |  |  |  |  |  |  |  |  |  |
|  |  |  |  |  |  |  |  |  |  |  |  |  |
|  | * Possible misidentification of the recombinant was suggested by the software, with the Italian isolate as possible actual recombinant | | | | | | | | | |  |  |
|  | ** Statistical analysis was performed at an highest acceptable P-value of 0.05 | | | | |  |  |  |  |  |  |  |
